# Three-Dimensionally Printed Temperature Sensors Based on Conductive PLA Materials

**DOI:** 10.3390/s25206348

**Published:** 2025-10-14

**Authors:** Agnese Staffa, Gašper Krivic, Mariachiara Tocci, Massimiliano Palmieri, Filippo Cianetti, Janko Slavič

**Affiliations:** 1 Department of Engineering, University of Perugia, Via Goffredo Duranti 93, 06125 Perugia, Italy; mariachiara.tocci@studenti.unipg.it (M.T.); massimiliano.palmieri@unipg.it (M.P.); filippo.cianetti@unipg.it (F.C.); 2 Faculty of Mechanical Engineering, University of Ljubljana, Aškerčeva 6, 1000 Ljubljana, Slovenia; gasper.krivic@fs.uni-lj.si (G.K.); janko.slavic@fs.uni-lj.si (J.S.)

**Keywords:** conductive PLA, 3D-printed sensors, temperature effect, thermal characterization

## Abstract

Recent innovations in thermoplastic extrusion 3D printing have promoted the development of functional materials, such as conductive composites, which lead the way to the creation of sensors embedded directly into printed structures. To this aim, this paper presents a feasibility study on the use of a commercial conductive PLA filament for the realization of a 3D-printed temperature sensor integrated into a thermoplastic structure. To this end, a series of experiments were conducted on 3D-printed samples to analyse the correlation between electrical resistance and temperatures. The results obtained show a clear and reproducible relationship between the two quantities, from which a useful function was derived to estimate the temperature from the resistance measurement. This study confirms the potential of conductive PLA as a low-cost and customisable solution for thermal monitoring and represents a step forward towards the integration of functional sensors through additive manufacturing.

## 1. Introduction

In recent years, the development of printed sensors has grown significantly, driven by advancements in material science that have opened new possibilities for fabricating embedded sensors using additive manufacturing (AM) techniques [1,2]. The increasing demand for objects with complex geometries that combine flexibility and mechanical strength—alongside the push towards the creation of smart structures—has propelled the research community toward innovative goals [3,4]. This momentum has led to the emergence of novel polymeric materials designed for the fabrication of electrically conductive composites [5,6]. In parallel, several studies have explored the integration of different materials through the inclusion of conductive particles in thermoplastic matrices such as carbon black, carbon fibre, carbon nanotubes, graphene, and conductive polymers [7,8]. These fillers, which are highly compatible with AM processes, enable the production of conductive filaments suitable for fused deposition modeling (FDM) [9]. Such filaments allow the creation of sensors or functional materials directly embedded in structures during the printing process itself [10].

These advancements pave the way for a broad range of applications for printed sensors, particularly in fields that require electrically conductive and flexible components. Notable examples include robotics [11], bioengineering [12], aerospace [13], and structural health monitoring [14,15,16].

Thermoplastic polylactic acid (PLA), in its conductive formulation (PLA filled with carbon black), has attracted growing attention in the field of printed sensors due to its suitability for the fabrication of functional sensors. This material has already been successfully employed in the development of devices capable of detecting various physical and chemical quantities, such as humidity, deformation, vibrations, and even certain thermal phenomena [17,18,19]. While these applications highlight the versatility of conductive PLA, recent observations have underscored the significant influence of temperature on its electrical resistance, opening new avenues for its use in thermal sensing [20].

Temperature-induced variations in the electrical resistance of conductive PLA, rather than being treated as a source of interference, can be effectively leveraged to develop dedicated temperature sensors. This temperature sensitivity opens up the possibility of employing thermoplastic conductive PLA as an active material for thermal sensing applications [21].

Previous research has investigated the possibility of using thermoplastic conductive PLA, processed using 3D printing technologies, to fabricate temperature sensors, highlighting its potential for thermal monitoring applications [22,23,24,25,26,27]. Additionally, other research has focused on characterizing the influence of temperature on the material’s electrical and physical behaviour, thereby laying the groundwork for its integration into functional thermal monitoring systems [28].

Building on these findings, Jeon et al. [22] examined the use of a Wheatstone bridge configuration to construct a thermometer capable of mapping temperature distributions across a surface within the 10 °C to 50 °C range, demonstrating a non-linear increase in resistance with rising temperatures; the same effect was already verified in [23] in the temperature range of 25–100 °C. In a complementary investigation [24], temperature tests were conducted between 20 °C and 90 °C, including both heating and cooling cycles. A notable difference in sensor response between the two phases was observed, revealing, for the first time, the hysteretic behaviour of the material. To provide a more detailed analysis, Stopforth [25] conducted a detailed experimental investigation into the electrical properties of conductive PLA—specifically resistance, resistivity, and the coefficient of linear expansion—as a function of temperature. The study established an empirical relationship between temperature and resistivity to characterize the material’s behaviour under thermal variations. Taking this a step further, Steckiewicz [26] examined the potential of using conductive PLA for temperature sensing on curved surfaces, ultimately deriving a hybrid resistance–temperature calibration curve over the range of 0 °C to 70 °C.

Collectively, these studies demonstrate the potential of thermoplastic conductive PLA for thermal sensing [27] while also revealing its non-linear and hysteretic response, particularly under thermal cycling. A non-linear relationship between resistance and temperature is consistently observed across the literature. This relationship is often well approximated by fourth-degree polynomial fitting [25,26].

Among the different sensing mechanisms observed, the variation of electrical resistance with temperature stands out as a particularly promising feature. This inherent property of conductive PLA suggests its potential use in the development of printed temperature sensors. Instead of being considered a secondary effect, the temperature-dependent change in resistance can be intentionally exploited to enable the direct detection of thermal variations.

These observations underscore the necessity for a more profound comprehension of the behaviour of conductive PLA under thermal loads. Such knowledge is critical for accurately characterising the material and enabling the development of reliable, high-performance temperature-printed sensors.

The objective of this research is to examine the impact of external temperature fluctuations on the electrical characteristics of commercial thermoplastic conductive PLA when utilised in sensor components exposed to electrical current. A fundamental step in evaluating the material’s aptitude for utilisation in sensor applications is to ascertain the extent to which temperature fluctuations impact its electrical resistance.

In order to address this, an experimental campaign was conducted to characterise the resistance-temperature behaviour of conductive PLA and to model this relationship using appropriate mathematical functions. The analysis focuses on two sensor configurations, both of which are fabricated by printing conductive PLA onto a PLA substrate, with resistance measurements performed under controlled thermal cycling.

To address limitations in previous studies, which primarily focused on single heating–cooling cycles within positive temperature ranges, this work expands the investigation to include both positive and negative temperature ranges, as well as multiple successive thermal cycles. This approach enables a more comprehensive characterization of the hysteretic behaviour and non-linear resistance–temperature relationship of conductive PLA, providing novel insights into its suitability for the fabrication of reliable 3D-printed temperature sensors.

This manuscript is organized in two parts: the first describes the fabrication of specimens with integrated sensors and the test procedure, while the second presents the analysis of the collected data.

## 2. Materials and Methods

### 2.1. Materials

The materials used in the following research activity belong to the category of thermoplastics used in additive manufacturing via fused filament deposition (FFD). Conventional Prusament PLA (Prusa Research, Prague, Czech Republic, 1.75 mm diameter [29]), a biodegradable thermoplastic material derived from polylactic acid, was used for the base structure, while conductive PLA was used for other parts, which is a filament made from a PLA matrix in which conductive particles are dispersed generally in the form of powders or carbon fibres, such as graphite, carbon black (CB), fibres, or carbon nanotubes [30]. The typical process involves mixing the PLA with the conductive filler by screw extrusion, followed by the homogenisation of the molten material to ensure uniform particle dispersion and subsequent extrusion into a filament with a controlled diameter (1.75 mm or 2.85 mm) suitable for the FFD technique [31]. In this activity, the ProtoPasta conductive PLA (Protopasta 3D Print Filament, Vancouver, WA, USA [32]) with a diameter of 1.75 mm and a concentration of 20% carbon black was used [33].

The inclusion of these particles permits the creation of continuous paths (percolating networks) that allow the passage of current, making it an electrically conductive material [34,35], for which its electrical resistance is sensitive to dimensional variations in the polymer: The thermal expansion or mechanical deformation of PLA modifies the distance between conductive particles, altering the continuity of the paths, causing variations in resistance depending on the temperature or stress applied, and leading to a variation in the resistivity of the material. This effect originates from the rearrangement of conductive paths within the polymer matrix under stress, and one general characteristic of conductive polymer composites is their non-linear resistance response to mechanical deformation [36].

Among the fillers used, carbon black is the most widely used due to its low cost, low density, good internal conductivity, and high surface/volume ratio [24]. The typical concentration of fillers varies between 20% and 25% by volume, a value that allows stable electrical conductivity to be achieved without excessively compromising mechanical properties. The distribution and morphology of these fillers determine not only electrical conductivity but also mechanical parameters such as strength and flexibility. In fact, mechanical tests have revealed that the tensile strength of conductive PLA reaches approximately 66% of that of pure PLA: The addition of fillers therefore improves electrical performance but reduces mechanical performance [37]. For this reason, the percentage of conductive particles is kept within optimal values in order to balance the two properties.

### 2.2. Methods

**Figure 1:**
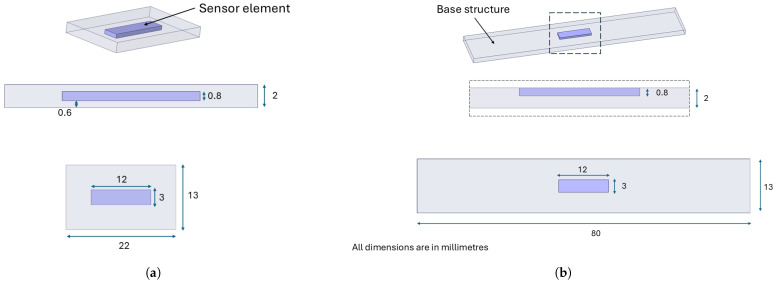
Shape and dimensions of the two types of sample: (**a**) embedded; (**b**) exposed.

The fabrication of all specimens was undertaken using the Prusa XL printer, Prague, Czech Republic [38], a device capable of utilizing two nozzles concurrently. This functionality enables the extrusion of two distinct materials, thereby facilitating the simultaneous printing of the sample and the sensor within a single printing process. To investigate the effect of temperature variations on material resistance, two different samples, as shown in Figure 1, were fabricated; the purple segment denotes the 3D-printed sensor element, whereas the grey segment corresponds to the base structure printed using a generic PLA filament.

The dimensions and shape of the sensor element remain constant in both configurations, ensuring that the only difference lies in its placement within the sample. The change in the length of the base structure for the exposed sensors shown in Figure 1b is due to the fact that a longer base structure allows for better placement of the sample in the climate chamber without affecting the results. We do not aim to verify the performance of the sensory element for different geometries, as this aspect has already been addressed in [16]. Instead, this study aims to analyse the effect of temperature on the material’s electrical behaviour and assess the feasibility of developing a functional temperature sensor, focusing, in particular, on how the element’s position within the sample influences its response under thermal fluctuations. As shown in Figure 1a, the sensors are fully embedded in the specimens, and they are located at a distance of 0.6 mm from both the top and bottom surfaces of the specimen. In contrast, as shown in Figure 1b, the specimen with the exposed sensor is not fully embedded in the test specimens, but the last layer of the sensor is aligned with the final layer of the test specimen, and the rest of the sensor is located inside. As previously stated, the specimens are identical, with the only variation being the position of the sensor element. Consequently, the printing parameters remain constant and are reported only once in Table 1.

**Figure 2:**
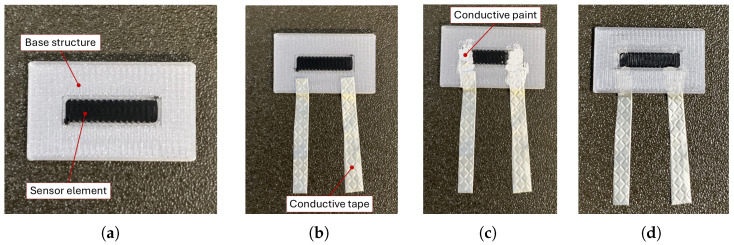
Sensor production phases: (**a**) printing of the first layer of conductive PLA; (**b**) inserting conductive tape; (**c**) inserting conductive paint layer; (**d**) continuous and the end of the printing process.

A clarification must be made regarding the description of the sensor manufacturing process (Figure 2), as pauses are inserted during the printing process to allow for the insertion of conductive tape and paint for the creation of the acquisition circuit.

**Figure 3:**
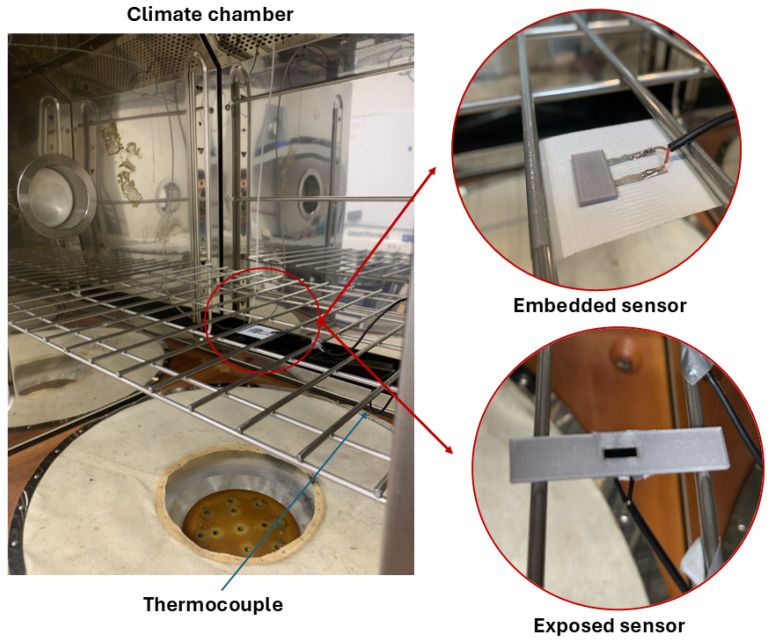
Experimental setup: positioning of the samples in the climatic chamber.

**Figure 4:**
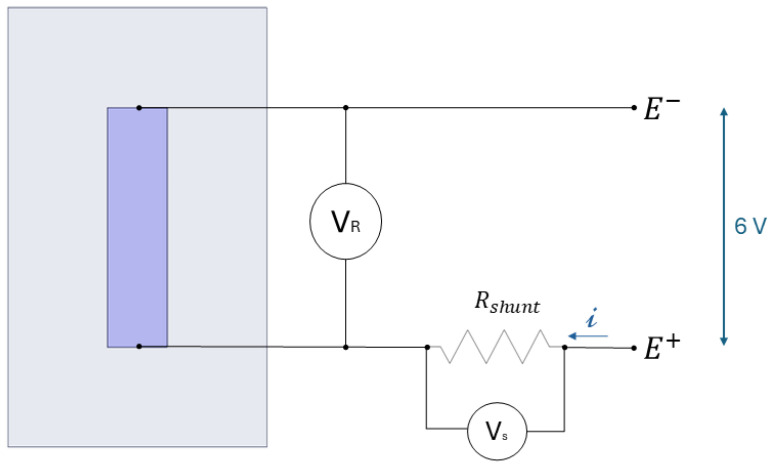
Schematisation of power supply and acquisition circuit.

The sensor element is composed of four layers, all of which were printed with the same printing direction set at 0°, as illustrated in Figure 2a. During the printing process, conductive tape is inserted between the second and third layers of the sensor (Figure 2b), and a layer of conductive paint is applied between the sensor and the conductive tape (Figure 2c). The tape is positioned so that it partially protrudes from the sample, as illustrated in Figure 2d. Once the printing process is complete, copper wires are soldered to the tape’s ends to create a strong electrical and mechanical connection to the measurement circuit, as shown in Figure 3. An ad hoc circuit was designed to simultaneously power the sensor with a 6-volt battery and to measure the voltage variation during the test. The schematisation of this is shown in Figure 4.

The acquisition circuit is thus composed of a fixed shunt resistor, with a resistance value that approximates that of the 3D-printed sensor. The current *i* flowing in the circuit through the two resistors in series is constant. This can be calculated using the voltage difference acquired at the ends of the shunt resistor, $V_{S}$, according to Ohm’s law. Once the current is determined, the sensor resistance can be obtained by measuring the voltage difference at the ends of the sensor, $V_{R}$. The resistance value can then be calculated using Equation (1).

(1) $$R_{sensor}=\frac{V_{R}}{V_{S}}\cdot R_{shunt}$$

### 2.3. Experiments

In order to analyse the influence of temperature on the change in resistance of 3D-printed sensors, a series of tests were conducted with the aid of a climate chamber, as shown in Figure 3.

**Figure 5:**
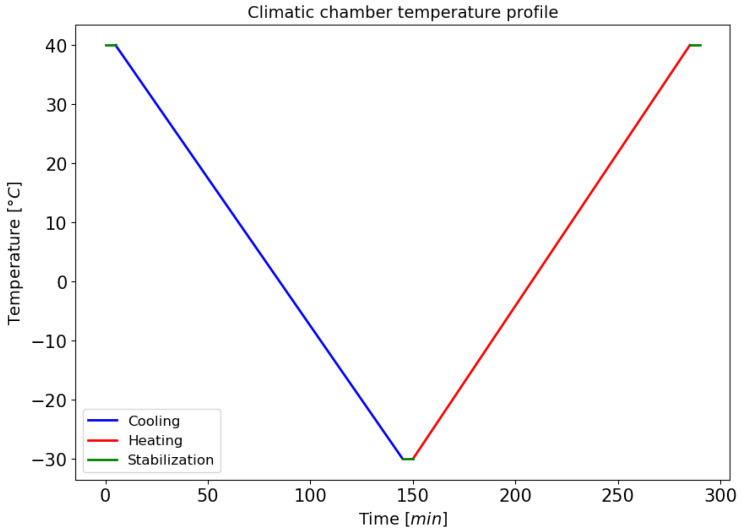
Imposed temperature profile during the test.

Inside the climatic chamber, the samples were placed on a grid, together with a centrally positioned thermocouple, to detect the temperature in the area adjacent to the samples. The power connections for the sensors and thermocouple exit the chamber through a special side hole and connect to the acquisition card. This setup enables the synchronized acquisition of resistance and temperature data for consistent subsequent processing. A constant sampling frequency equal to 1 Hz was used in each test. The experimental procedure involved subjecting the samples to a temperature profile initiated at 40 °C, followed by a cooling process to −30 °C at a rate of 0.5 °C/min. Subsequent to this phase, the temperature is maintained at a constant level for a period of five minutes prior to the initiation of a new heating phase (see Figure 5).

The upper temperature limit was deliberately chosen to remain below the glass transition temperature of conductive PLA (55–60 °C) [39]. Beyond this temperature, the material undergoes a significant change in its mechanical and electrical properties [23,40,41]. The introduction of additional variables would be inevitable if this threshold were to be exceeded. This would make it difficult to isolate and study the effect of temperature solely on the sensor’s response. Consequently, maintaining this limit ensures that any observed behaviour can be attributed to sensor performance rather than to intrinsic changes in the material itself.

Conversely, the lower limit was set at −30 °C to ascertain the feasibility of employing conductive PLA in low-temperature environments, such as aerospace applications [13,20]. Despite the fact that, at these lower temperatures, the material becomes more brittle and mechanically unstable [40], it remains scientifically relevant to investigate how such conditions affect the sensing performance. This is especially true when considering the limited existing literature on the behaviour of conductive PLA at sub-zero temperatures.

The temperature tests were repeated multiple times (four times in preliminary tests and twelve times in the longer test, as detailed in Table 2) for each configuration, (with the relative humidity maintained constant at 50%, in the range where the chamber is able to control the humidity level (0 °C–70 °C). Throughout the entire duration of the tests, both the voltage across the resistive elements and the signal from the thermocouple were continuously acquired.

Table 2 provides a summary of the tests performed, reporting the type of sensor used (embedded or exposed), the initial resistance of each sensor measured immediately after the printing process (expressed in Ohms and indicated in the table as “Resistance Sensor”), and the number of repetitions of the thermal cycle applied for each test.

Tests 1 and 2 were performed on embedded and exposed sensors, respectively, while test 3 was performed on both types of sensors, the type of which is reported in brackets in Table 2.

The preliminary tests were carried out separately on embedded and exposed sensor configurations, each subjected to four thermal cycles. The objective was to assess the feasibility of sensor operation in both configurations. Following these initial trials, an additional test was performed to further evaluate the sensor response and investigate the correlation between temperature variation and resistance change. The subsequent sections present and discuss the results obtained from these experiments.

### 2.4. Analysis of Results

This section illustrates the methodology used to evaluate the data obtained from the tests conducted. The analysis procedure is described below in a simplified sequence of steps:

1. Calculations of the normalized variation in resistance relative to the initial value are essential due to inherent differences among the samples. These variations may arise from inconsistencies in the manufacturing process, the dimensions of the silver tape (which can vary since it is cut by hand), and the positioning of the tape on the conductive PLA layer. Normalization of the resistance allows the identification of the general behaviour of the sensor and enables a reliable comparison between different tests, effectively evaluating the repeatability of the measurements. Resistance is normalized based on the voltage data acquired during the test using the following equation [42]:
  (2) $$\frac{\Delta R}{R}=\frac{R_{i}-R_{0}}{R_{0}}$$
  where $R_{i}$ is the instantaneous resistance, and $R_{0}$ is the initial resistance value measured after the printing and wire soldering phase, as indicated in Table 2;
2. Identification of individual temperature cycles within the data and plotting the corresponding resistance variations;
3. Exponential curve fitting for both the heating (ascending) and cooling (descending) phases in order to derive a model describing the correlation between resistance change and temperature [26];
4. Determination of a reference value to quantify the deviation between the experimental data and the fitted curve;
5. Estimation of the associated fitting error.

## 3. Results

This section presents the results of the conducted tests. The discussion begins with the preliminary experiments (comprising four consecutive temperature cycles), followed by an analysis of the longer-duration tests (consisting of twelve consecutive cycles).

This procedure was applied to each sensor and each test individually. For clarity and brevity, results from a representative sensor for each test are presented, followed by a comparative summary of the results across all tests.

### 3.1. Preliminary Test

**Figure 6:**
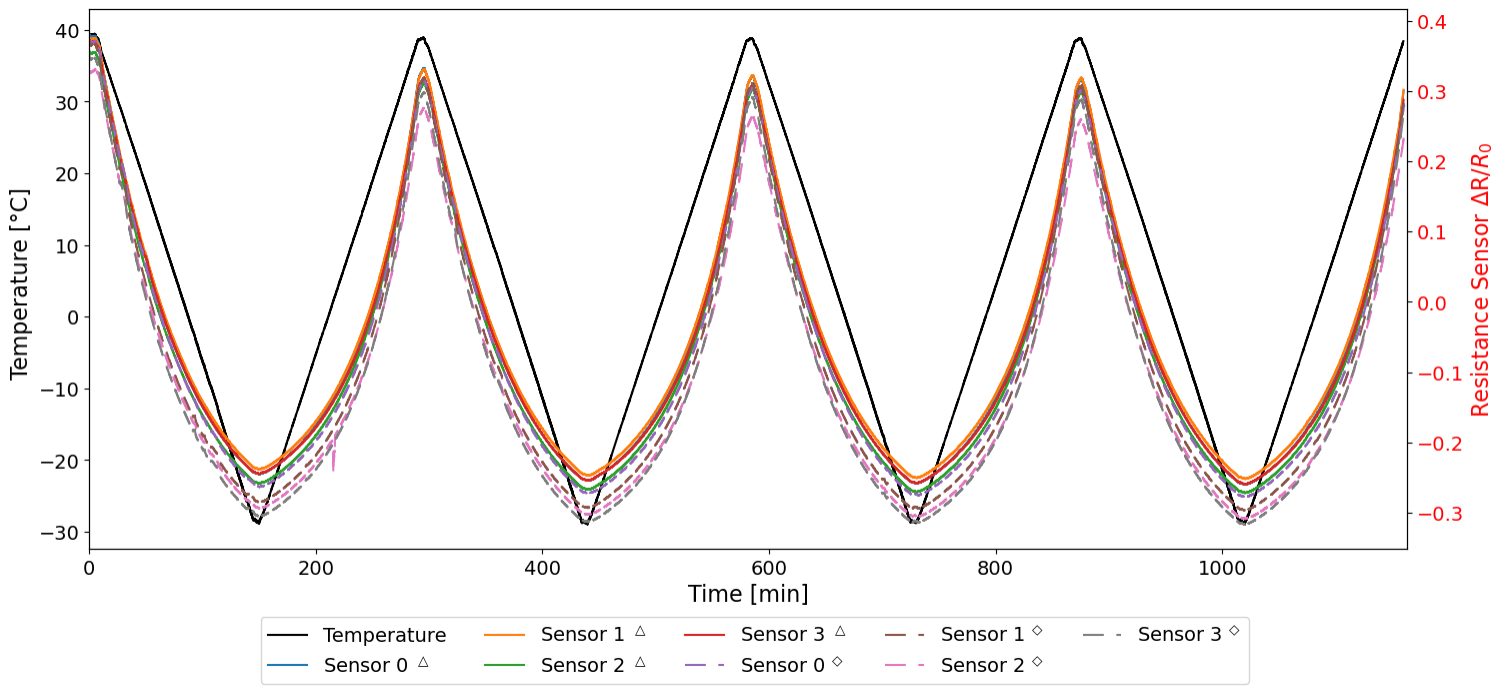
Resistance and temperature time histories acquired during TEST 1 and TEST 2.

From the first two preliminary tests, as summarized in Table 2, the normalized resistance variations with respect to the initial value were computed, as defined in Equation (2). The results are illustrated in Figure 6, which show the time history of the resistance during the thermal cycles for the embedded (solid line) and exposed (dashed line) sensors, respectively.

In Figure 6, the resistance is normalized to its initial value, as detailed in Section 2.4, to account for the intrinsic differences of the specimens manufactured by additive manufacturing. As demonstrated in Figure 6, the behaviour of both sensors is similar, with the ascending (heating) and descending (cooling) trends in the resistance variation of all sensors being closely aligned. The normalized data confirms that measurements derived from disparate samples and tests are demonstrably repeatable.

**Figure 7:**
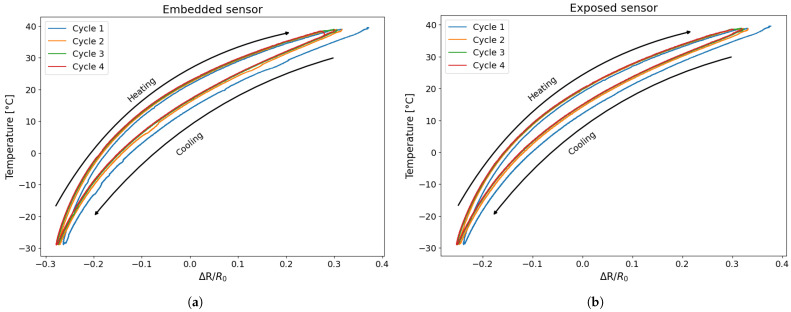
Temperature variation as a function of resistance change: (**a**) embedded sensor; (**b**) exposed sensor.

In Figure 7, the variation in resistance is plotted as a function of the temperature recorded during the test. It can be observed that the resistance variation due to temperature reaches approximately 60%, as previously reported in the literature [23], and that the trend is not linear but rather exhibits a quadratic or parabolic behaviour [20,22,25,26]. A distinct behaviour between the heating and cooling phases can be observed from Figure 7, as the resistance curves do not overlap; instead, they exhibit a separation. Specifically, for the same resistance change, the corresponding temperatures differ, indicating the hysteretic behaviour of the sensor. This phenomenon is consistent with expectations based on the intrinsic thermal and mechanical properties of the PLA-based material [23], as well as with observations reported in previous studies [20,24].

Two fundamental aspects emerge from Figure 7: The resistance change during the cooling phase is systematically lower than that of the heating phase, indicating a clear hysteretic behaviour in the sensor’s response to temperature variations, a phenomenon that is already highlighted by [24,27]; furthermore, for both sensors, the first cycle is larger than the others, and starting from the second cycle, the resistance–temperature relationship remains constant. To clarify this concept further, an estimation of the areas of the individual hysteresis cycles was carried out for both types of sensor. Table 3 shows the area for the preliminary tests.

**Figure 8:**
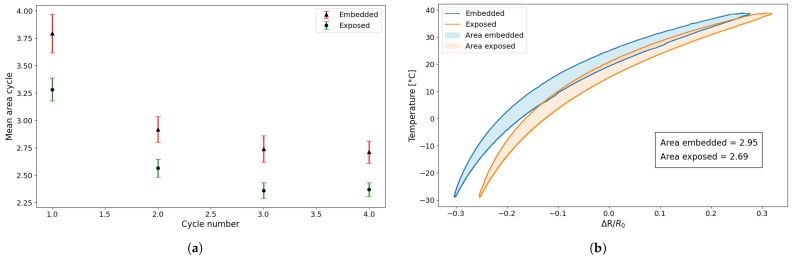
Comparison of area: (**a**) mean and standard deviation between embedded and exposed sensor; (**b**) second cycle of an embedded and an exposed sensor.

In Figure 8a, the average area calculated on all samples tested for each cycle is plotted with the relative standard deviation, while Figure 8b shows the superimposition of a single cycle of an embedded sensor and an exposed sensor to directly compare their response behaviour.

The first element that emerges from Figure 8 and from Table 3 is that the behaviour of the area for the two types of sensors is very similar, although the embedded sensor has an area that is about 13% larger than the exposed one. Furthermore, while the amplitude of the hysteresis loop is almost the same, Figure 8b clearly highlights a difference in the relative displacement between the two loops. The embedded sensor shows a backward shift of approximately 20% compared to the exposed sensor due to the thermal conductivity of the material. In fact, the embedded sensor is affected by the heating of the entire sample, which delays reaching thermal equilibrium with the surrounding environment and causes it to measure temperatures that are higher than ambient temperatures.

The second element, clearly distinguishable from Figure 8a and Table 3, is the significant difference between the first cycle and the subsequent ones for both sensor types. Specifically, the first thermal cycle demonstrates a greater resistance variation compared to the following cycles. From the second cycle onwards, however, the heating and cooling phases become more consistent and tend to overlap more closely. This behaviour suggests that the sensor undergoes a form of stabilisation after the initial exposure to temperature variation, resulting in improved repeatability. Consequently, the initial cycle was excluded from the analysis, with the study commencing from the second cycle.

Despite this hysteresis, an approximation of the experimental data was performed to obtain a representative fitting curve of resistance as a function of temperature in order to evaluate the accuracy of the 3D-printed sensor in tracking temperature variations. Although this approach does not explicitly account for hysteresis, it nevertheless enables an estimation of the measurement error associated with the utilisation of the sensor as a temperature probe. This simplification is imperative in practical applications, where it is not always feasible to ascertain a priori whether the temperature is increasing or decreasing.

**Figure 9:**
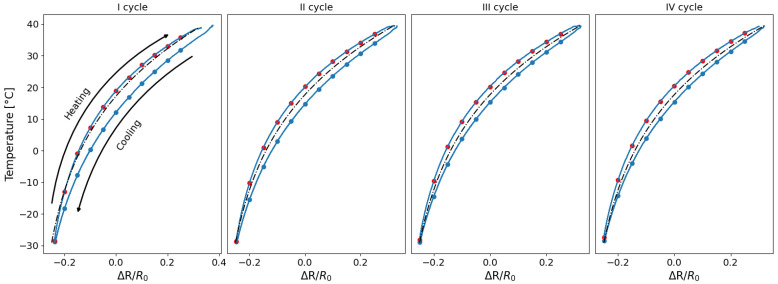
Temperature variations as a function of resistance changes with the relative fitting curve.

The selection of an exponential fitting curve was determined to be the most appropriate for the approximation process, as it offered the optimal alignment with the experimental data, as illustrated in Figure 9. The fitting curve was generated using all data points from the second cycle onwards across all temperature cycles. The values used to evaluate the error of the experimental data points, as shown in Figure 9, were selected at regular intervals within the range of resistance variations observed during the experimental tests.

**Figure 10:**
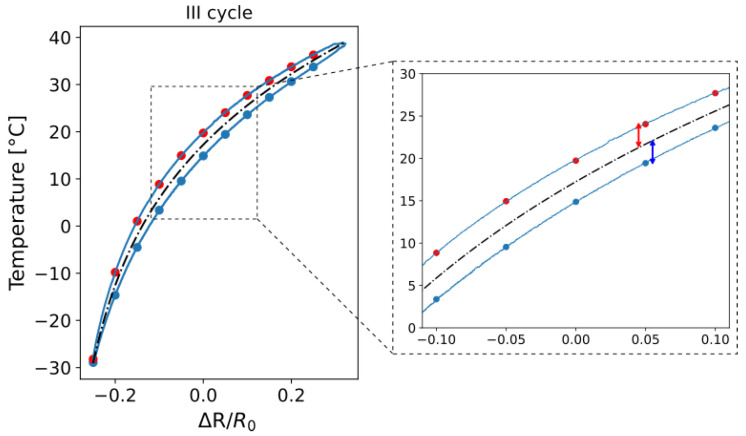
Procedure for the evaluation of the difference between the experimental data and the corresponding point on the fitting curve.

Following the determination of the fitting curve, the associated error was calculated as the deviation between the experimental data and the fitted values, as illustrated in Figure 10. Specifically, the error was determined by evaluating the difference between each experimental data point—both from the heating and cooling phases—and the corresponding point on the fitting curve. These deviations are visually represented in Figure 10 by the red and blue arrows, which correspond to the heating and cooling curves, respectively.

**Figure 11:**
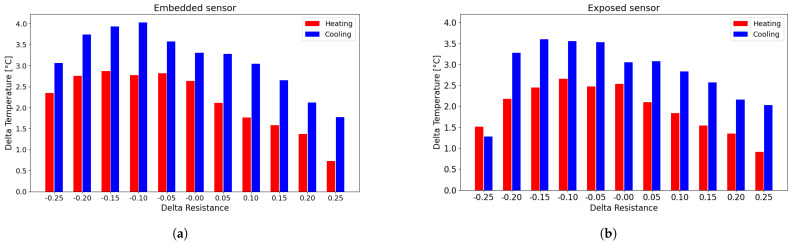
Histogram of the difference between the experimental data and the corresponding point on the fitting curve: (**a**) embedded sensor; (**b**) exposed sensor.

For each resistance change value that was selected, the difference between the heating and cooling curves was calculated separately for each cycle (second, third, and fourth) from the fitting curve. Given the similarity of the errors obtained between the different cycles, these were averaged for each value that was selected over each cycle and for the number of sensors of the same type. This procedure was then performed for all sensors tested in the preliminary tests and then averaged. Figure 11 shows the overall errors for both embedded and exposed sensors and represents the averaged error between the heating and cooling cycles with respect to the fitting curve.

The error associated with using a single interpolation curve is less than 4 °C per measurement for embedded sensors and approximately 3.5 °C for exposed sensors. As expected, the error with exposed sensors is lower in comparison to the embedded sensor. This is a direct consequence of the hysteresis cycle areas of exposed sensors in comparison to the embedded sensors, as shown in the Figure 8 and in Table 3. Therefore, the distance between the two branches is smaller, as is the distance with the approximation curve.

Subsequent to this, once the fitting error had been quantified, the analysis concentrated on evaluating the repeatability of the fitted curves obtained for the tested sensor. In order to assess this, the individual fitting curves corresponding to each of the tested sensors were plotted on a single graph. This facilitated a comparative analysis of the dispersion of the individual curves with respect to the mean trend, thereby providing insight into the consistency of sensor behaviour across different samples.

The mean curve and its corresponding standard deviation were then calculated based on the fitted curves obtained from the experimental data for each specimen.

**Figure 12:**
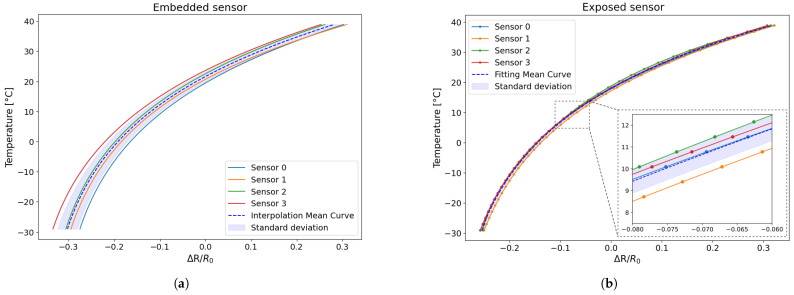
Comparison between the individual interpolation curves corresponding to each of the tested sensors with the global mean curve and the corresponding standard deviation: (**a**) embedded sensor; (**b**) exposed sensor.

As is visible in Figure 12, the data dispersion for the embedded sensors ranges between approximately 2.1% and 2.5%, whereas the exposed sensors exhibit significantly lower variability, with standard deviation values between 0.3% and 0.6%.

The findings suggest that the derived fitting curves are indicative of the overall sensor behaviour. Consequently, these mean curves will serve as reference benchmarks in subsequent tests, thereby enabling the evaluation of sensor performance consistency and the repeatability of their thermal response.

### 3.2. Results of the Long Cycle

Following the confirmation of the functionality of the sensors, a third test was conducted. This test involved the application of a longer sequence of 12 temperature cycles, with two exposed and two embedded sensors.

**Figure 13:**
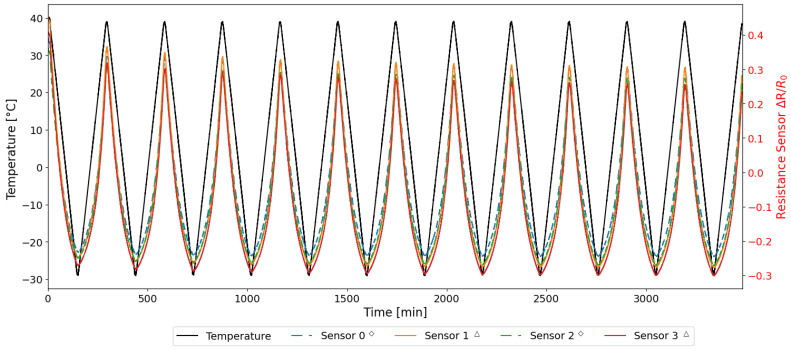
Resistance and Temperature time histories acquired during the TEST 3.

As illustrated in Figure 13, the resistance and temperature variations recorded during the entirety of the test are presented. Figure 13 shows that both type of sensors manifest a behaviour that is consistent with the trends observed in the previous tests. Consequently, the same analytical procedure was applied to these cycles, and only the resulting data are presented and discussed in the following sections.

**Figure 14:**
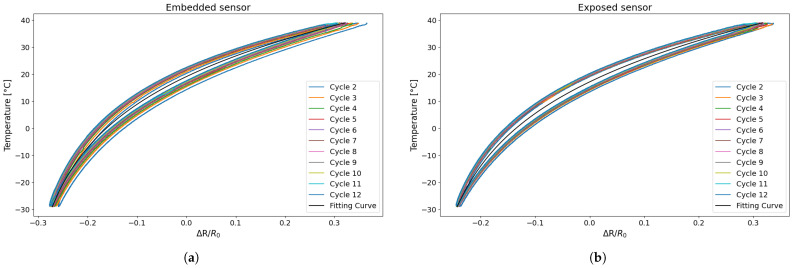
Temperature variation as a function of resistance change with the relative fitting curve for TEST 3: (**a**) embedded sensor; (**b**) exposed sensor.

First, the approximation curves for the embedded and separately exposed sensors were determined considering the second to last cycle. Figure 14 shows an example of an exposed sensor and an embedded sensor with the relative cycles and calculated approximation curve.

**Figure 15:**
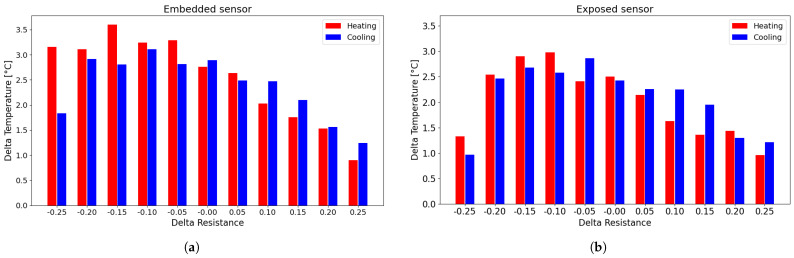
Histogram of the difference between the experimental data and the corresponding point on the fitting curve for the TEST 3: (**a**) embedded sensor; (**b**) exposed sensor.

Subsequently, the differences between the experimental values and the fitted curve were calculated to evaluate the deviation from the heating and cooling curves, as shown in Figure 15.

As shown in Figure 15, the errors associated with using the fitting curve instead of the actual measured data are approximately 2 °C for the exposed sensors and around 3 °C for the embedded sensors, which is significantly smaller than in the earlier cases. This reduction is due to the progressive narrowing of the hysteresis loops as the number of cycles increases, leading to smaller discrepancies between heating and cooling phases and, consequently, between the experimental data and the fitted curve.

**Figure 16:**
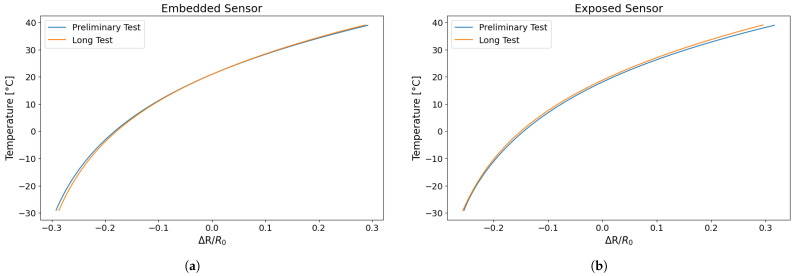
Comparison of the fitting curve of different tests of the trend in temperature as function of resistance change: (**a**) embedded sensor; (**b**) exposed sensor.

The fitting curves obtained from the preliminary test (TEST 1 and TEST 2) and the longer test (TEST 3) were compared in order to evaluate the reproducibility of the sensor behaviour between the various samples and conditions. As illustrated in Figure 16, the mean fitting curves obtained from the shorter and the longer tests are presented.

As shown in Figure 16, the mean fitting curves from the shorter and longer tests are almost superimposable, indicating that the sensor’s behaviour remains constant even during prolonged testing. Furthermore, it is noteworthy that sensors printed and tested at different times exhibit highly overlapping interpolation curves, which is indicative of good reproducibility.

The following equations (Equations (3) and (4)) represent the average fitting curves obtained from Test 3 for both types of sensor, which allow the temperature to be estimated from a known resistance change. This is a useful function when the sensor is employed for temperature monitoring.

Embedded sensor:

(3) $$T\left(\frac{\Delta R}{R}\right)=25.8\cdot e^{1.492\frac{\Delta R}{R}}-5.312\cdot e^{-7.555\frac{\Delta R}{R}}$$

Exposed sensor:

(4) $$T\left(\frac{\Delta R}{R}\right)=23.15\cdot e^{1.926\frac{\Delta R}{R}}-4.86\cdot e^{-8.64\frac{\Delta R}{R}}$$

Finally, to demonstrate the reproducibility of the results, a comparison was made between the various cycles of the sensors tested in the preliminary tests and in the long test. Table 4 shows the values of the areas of the individual hysteresis cycles obtained for the sensors tested in the long test.

**Figure 17:**
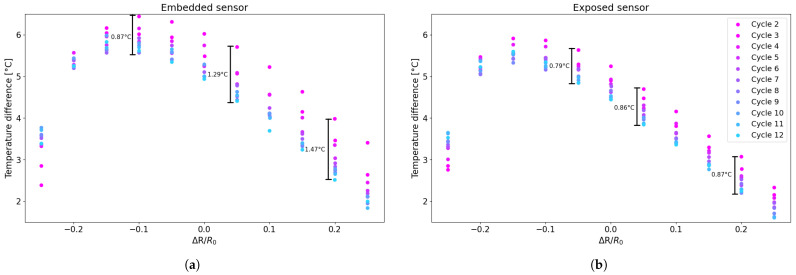
Differences between ascent and descent cycles for the following: (**a**) embedded sensor; (**b**) exposed sensor.

Furthermore, Figure 17 shows the absolute difference in resistance variation across all temperature cycles for specific resistance variation values. For predefined resistance values previously utilised in the error assessment detailed in Section 3.1, the difference between the corresponding values on the heating and cooling curves was calculated. With reference to Figure 10, this is equivalent to the variation between the red and blue data points. The resulting data were arranged into a single graph (Figure 17) to observe the overall trend as the number of cycles increased, showing the absolute differences between the heating and cooling phases for each cycle.

As can be seen in Figure 17, the difference between the heating and cooling curves gradually decreases as the number of cycles increases, decreasing by approximately 1° for exposed sensors and approximately 1.5° for embedded sensors. This result is also confirmed by the fact that, as can be seen in the table, the area of the hysteresis cycles decreases as the number of cycles increases, so the distance between temperatures for a relative resistance variation value decreases.

To complete the analysis of the repeatability of the behaviour of the printed sensors, the averages of the areas of each cycle of the two types of sensors tested were compared for the two tests conducted (preliminary and long). Comparing the average area values of the first four hysteresis cycles, these differ by 1–2% for exposed sensors and 5–7% for embedded sensors in the first four cycles, demonstrating that although the sensors are printed and tested using the same procedure but performed at different times, the behaviour is repeated. It can also be noted that the hysteresis response of the sensor becomes less pronounced over time, gradually decreasing and stabilising.

**Figure 18:**
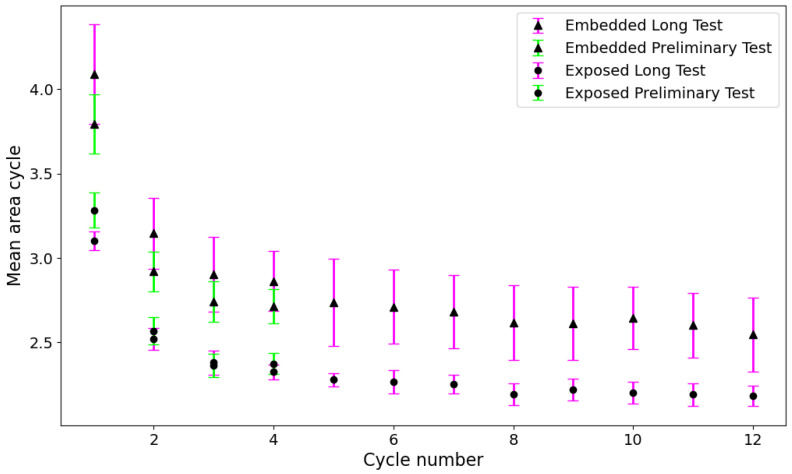
Comparison of the average area under the cycles for all sensors in Tests 1, 2 and 3.

Another important observation from the graph (Figure 18) is that the values are higher during the initial cycles but then decrease and stabilise after about the eighth cycle. Furthermore, the dispersion of values obtained from the embedded sensors across all analyses is consistently greater than that from the exposed sensors. As demonstrated by the graphs and the corresponding equations, some differences in behaviour can be observed between the two sensor configurations. Nevertheless, the overall trend in resistance variations remains consistent. The minor offset detected between the curves of the embedded and exposed sensors can be attributed to the elevated influence of the specimen’s thermal mass on the embedded configuration, resulting in a slightly divergent thermal response.

## 4. Conclusions

In this study, the thermal–electrical behaviour of a commercial conductive PLA filament (ProtoPasta) was investigated with the aim of evaluating its feasibility for temperature sensing applications through additive manufacturing. Sensors were fabricated via fused filament deposition directly onto PLA substrates, confirming the possibility of embedding functional sensing elements into thermoplastic structures during the printing process, with the additional advantages of low cost and design flexibility. The main findings of the work can be summarized as follows:

- The resistance of conductive PLA shows a clear and repeatable dependence on temperature. Although the material exhibits hysteresis during thermal cycling, its response remains stable and consistent across repeated loadings. A comparison between embedded and exposed sensors indicated that exposed sensors generally display lower variability and reduced hysteresis, resulting in smaller deviations in measured values.
- A functional relationship between resistance and temperature was identified through curve fitting. Within the scope of this feasibility study, the derived model showed deviations of about 3 °C between predicted and measured values. This margin should not be considered as a definitive sensor accuracy but rather as an indication of the potential of conductive PLA for temperature sensing. More complex models would allow for a better estimation of the temperature including also the hysteretic behaviour of the sensor.
- A distinctive contribution of this work lies in its extended characterization beyond the positive temperature ranges commonly investigated in the literature, including multiple heating and cooling cycles across both positive and sub-zero conditions. This novelty demonstrates the potential of conductive PLA sensors for applications in environments with fluctuating or harsh thermal conditions.

Overall, this study highlights the potential of conductive PLA as a viable material for the realization of 3D-printed, integrated, and cost-effective thermal sensors, while also identifying challenges—such as hysteresis—that motivate further research and optimization.

## Figures and Tables

**Table 1:** Printing parameters.

Parameters	Prusament PLA	Conductive PLA (Protopasta)
Layer height [mm]	0.2	
Infill density	100%	
Fill angle [°]	0	
Infill pattern	Rectilinear	
Infill layer thickness [mm]	0.2	
Nozzle printing temperature [°C]	220	
Bed printing temperature [°C]	60	
Infill extrusion width [mm]	0.45	0.42
Bottom solid layers [num]	3	0
Perimeters [num]	2	0
Top solid layers [num]	5	0
Printing speed [mm/s]	170	120

**Table 2:** Summary of the performed test.

	Preliminary Test		Long Test
**DATA**	**TEST 1** **EMBEDDED**	**TEST 2** **EXPOSED**	**TEST 3**
Resistance Sensor 0	600 Ω	431 Ω	410 Ω (Exposed)
Resistance Sensor 1	630 Ω	446 Ω	630 Ω (Embedded)
Resistance Sensor 2	550 Ω	435 Ω	470 Ω (Exposed)
Resistance Sensor 3	575 Ω	410 Ω	650 Ω (Embedded)
Number cycles	4	4	12

**Table 3:** Area cycle of embedded and exposed sensor preliminary test.

	Number Cycle	Embedded Sensor				Exposed Sensor			
Number Sensor		**Cycle I**	**Cycle II**	**Cycle III**	**Cycle IV**	**Cycle I**	**Cycle II**	**Cycle III**	**Cycle IV**
Sensor 0		3.89	2.99	2.81	2.76	3.16	2.55	2.32	2.32
Sensor 1		3.49	2.72	2.55	2.55	3.20	2.56	2.36	2.35
Sensor 2		3.86	2.95	2.72	2.71	3.33	2.46	2.29	2.32
Sensor 3		3.93	3.01	2.88	2.83	3.43	2.69	2.47	2.47
**Mean**		3.79	2.92	2.74	2.71	3.28	2.57	2.36	2.37
**Standard Deviation**		0.17	0.12	0.12	0.10	0.17	0.11	0.12	0.10

**Table 4:** Areas of the cycle of the long embedded and exposed sensor test.

	Cycle	I	II	III	IV	V	VI	VII	VIII	IX	X	XI	XII
Sersor													
**Embedded**													
Sensor 1		3.79	2.93	2.68	2.68	2.48	2.48	2.47	2.40	2.40	2.45	2.41	2.32
Sensor 3		4.38	3.35	3.13	3.04	2.99	2.93	2.89	2.84	2.83	2.82	2.79	2.76
**Mean**		4.08	3.15	2.90	2.86	2.74	2.71	2.68	2.62	2.61	2.64	2.60	2.54
**Standard** **Deviation**		0.29	0.21	0.22	0.17	0.26	0.22	0.22	0.22	0.21	0.18	0.19	0.22
**Exposed**													
Sensor 0		3.16	2.59	2.45	2.37	2.32	2.33	2.31	2.25	2.28	2.27	2.25	2.24
Sensor 2		3.05	2.45	2.30	2.28	2.24	2.19	2.19	2.13	2.15	2.13	2.12	2.12
**Mean**		3.11	2.52	2.38	2.32	2.28	2.26	2.25	2.19	2.21	2.20	2.19	2.18
**Standard** **Deviation**		0.056	0.066	0.07	0.043	0.038	0.068	0.056	0.064	0.065	0.065	0.068	0.061

## Data Availability

Data are available upon request.
